# P-1058. Quantifying the Economic Consequences of Low Influenza Immunization Rates in the U.S

**DOI:** 10.1093/ofid/ofaf695.1253

**Published:** 2026-01-11

**Authors:** Joaquin F Mould-Quevedo, Van Nguyen

**Affiliations:** CSL Seqirus Inc., Summit, NJ; VHN Consulting, Montreal, Quebec, Canada

## Abstract

**Background:**

Influenza is a persistent public health concern in the United States, causing considerable morbidity and mortality each year. Despite the widespread availability of vaccines, low immunization rates contribute to the heightened economic burden of influenza, primarily through increased direct medical costs. This study employs advanced economic modeling to quantify the direct medical expenses associated with suboptimal vaccination coverage in the U.S. across different flu immunization rate scenarios. Influenza remains a significant public health and economic concern in the United States, causing substantial morbidity, mortality, and financial burden annually. Despite widely available vaccines, persistently low immunization rates lead to increased direct medical costs. This study utilized economic modeling to quantify direct medical expenses linked to inadequate influenza vaccination coverage.

**Methods:**

We employed a dynamic, age-stratified transmission model to estimate influenza-associated costs under varying immunization rate scenarios (25%, 30%, 35%, and 40%). Two distinct flu seasons were modeled: low incidence (2011-2012) and high incidence (2017-2018). Cost outcomes included total influenza-related outpatient and inpatient expenditures. Vaccine effectiveness (VE) was averaged from CDC reports over the past ten seasons (42%). The analysis assumed universal quadrivalent influenza vaccination across all age groups.

**Results:**

At the current U.S. influenza vaccination rate (∼35%), total estimated influenza-associated medical costs during a high-incidence season amounted to US$15,463M for outpatient care and US$28,331M for inpatient care. In a low-incidence season, these costs were US$8,042M (outpatient) and US$12,411M (inpatient). Achieving the Healthy People 2030 target vaccination rate (70%) would yield significant economic savings: approximately US$27,965M during high-incidence seasons and US$19,865M during low-incidence seasons.

**Conclusion:**

Quantifying the economic impact of low influenza vaccination rates underscores the urgency of increasing vaccination coverage. Improving immunization rates to recommended targets represents a highly cost-effective approach, significantly reducing direct medical expenditures.

**Disclosures:**

Joaquin F. Mould-Quevedo, PhD, CSL Seqirus: Employee|CSL Seqirus: Employee|CSL Seqirus: Stocks/Bonds (Private Company)|CSL Seqirus: Stocks/Bonds (Private Company)|CSL Seqirus Inc.: Employee|CSL Seqirus Inc.: Stocks/Bonds (Private Company) Van Nguyen, PhD, CSL Seqirus: Advisor/Consultant|CSL Seqirus: Advisor/Consultant|CSL Seqirus Inc.: Advisor/Consultant

